# Sustained Oxidative Stress Causes Late Acute Renal Failure via Duplex Regulation on p38 MAPK and Akt Phosphorylation in Severely Burned Rats

**DOI:** 10.1371/journal.pone.0054593

**Published:** 2013-01-17

**Authors:** Yafei Feng, Yi Liu, Lin Wang, Xiaoqing Cai, Dexin Wang, Kaimin Wu, Hongli Chen, Jia Li, Wei Lei

**Affiliations:** 1 Department of Orthopedic Surgery, Xijing Hospital, Fourth Military Medical University, Xi'an, China; 2 Department of Oral Implantology, School of Stomatology, Fourth Military Medical University, Xi'an, China; 3 Department of Physiology, School of Basic Medical Sciences, Fourth Military Medical University, Xi'an, China; 4 Department of Urology, Xijing Hospital, Fourth Military Medical University, Xi'an, China; 5 Department of Toxicology, Fourth Military Medical University, Xi'an, China; College of Tropical Agriculture and Human Resources, University of Hawaii, United States of America

## Abstract

**Background:**

Clinical evidence indicates that late acute renal failure (ARF) predicts high mortality in severely burned patients but the pathophysiology of late ARF remains undefined. This study was designed to test the hypothesis that sustained reactive oxygen species (ROS) induced late ARF in a severely burned rat model and to investigate the signaling mechanisms involved.

**Materials and Methods:**

Rats were exposed to 100°C bath for 15 s to induce severe burn injury (40% of total body surface area). Renal function, ROS generation, tubular necrosis and apoptosis, and phosphorylation of MAPK and Akt were measured during 72 hours after burn.

**Results:**

Renal function as assessed by serum creatinine and blood urea nitrogen deteriorated significantly at 3 h after burn, alleviated at 6 h but worsened at 48 h and 72 h, indicating a late ARF was induced. Apoptotic cells and cleavage caspase-3 in the kidney went up slowly and turned into significant at 48 h and 72 h. Tubular cell ROS production shot up at 6 h and continuously rose during the 72-h experiment. Scavenging ROS with tempol markedly attenuated tubular apoptosis and renal dysfunction at 72 h after burn. Interestingly, renal p38 MAPK phosphorylation elevated in a time dependent manner whereas Akt phosphorylation increased during the first 24 h but decreased at 48 h after burn. The p38 MAPK specific inhibitor SB203580 alleviated whereas Akt inhibitor exacerbated burn-induced tubular apoptosis and renal dysfunction. Furthermore, tempol treatment exerted a duplex regulation through inhibiting p38 MAPK phosphorylation but further increasing Akt phosphorylation at 72 h postburn.

**Conclusions:**

These results demonstrate that sustained renal ROS overproduction induces continuous tubular cell apoptosis and thus a late ARF at 72 h after burn in severely burned rats, which may result from ROS-mediated activation of p38 MAPK but a late inhibition of Akt phosphorylation.

## Introduction

As a major complication of severe burn injury, acute renal failure (ARF) complicates between 15% and 40% of the admissions in burn intensive care units (ICUs) [1], [2]. The onset of renal insufficiency predicts remarkably unfavorable prognosis of the burn patient as a high mortality rate of around 80% [3]. Two different forms of ARF have been described in burned patients: early and late ARF, depending on the time of onset [4]: while the early ARF occurs during the first few days and is related to hypovolemia, the late one, beginning more than five days postburn, has a more complex pathogenesis correlating with sepsis and multiorgan failure. Burn patients with late ARF are believed to be associated with worse prognosis and higher mortality [5], [6]. Although inflammation and apoptosis are reported to contribute to the development of late ARF in burn patients [7], [8], the pathogenesis is multifactorial and still poorly understood in many respects. Better understanding of the pathophysiology of delayed ARF during burn shock and searching for effective therapeutic strategies are critical to improve outcomes of severely burned patients.

Mitochondrial and cellular reactive oxygen species (ROS) have been well accepted as common manifestation and important inflammatory mediator which ultimately cause local and distant pathophysiological effects under several pathological conditions such as diabetes, sepsis and burn injury [9], [10], [11]. Because the abundance of polyunsaturated fatty acids make the kidney an organ particularly vulnerable to oxidative stress, ROS becomes a wide range mediator of renal impairments in renal diseases [12], [13]. Several recent studies indicate that under chronic conditions such as diabetic nephropathy, excess ROS leads to systemic apoptotic response and kidney injury [14]. The interaction of oxidative stress and apoptotic pathway is reported to be implicated in the development and progression of renal dysfunction [15]. Although clinical evidence showed that ROS production in affected tissue increased in pathophysiological events observed in burn patients [16], [17], [18], the role of ROS in pathogenesis of late ARF postburn remains unclear.

Prior studies have indicated that ROS mediated the activation of the mitogen-activated protein kinase (MAPK) signaling proteins, including extracellular signal-regulated kinase (ERK), p38 MAPK, and Jun N-terminal kinase (JNK), which are involved in growth arrest and apoptosis in nephropathy [19]. It is well known that cellular stresses upregulate JNK and p38 MAPK which play an important role in cell apoptosis and renal pathologies, while growth factors and trauma stimuli activate ERK which offers anti-oxidative effect and cell protection [20], [21]. In addition, the protein kinase B (Akt) signaling is reported to be closely linked with cell survival in burn [22], trauma [23] and ischemia/reperfusion injury [24]. Although Akt has been considered to be activated by oxidative stress and worked as a cellular antioxidant defense, it is reported that the high levels of ROS may block the activation of Akt pathway [25], [26], [27]. However, it still needs to further investigate the role of MAPK and Akt pathway in burn-induced late ARF and their relationship with ROS.

Therefore, the aims of the present study were 1) to determine whether ROS played a role in burn-induced tubular cell apoptosis and late ARF, and, if so, 2) to investigate the potential relationship between ROS and MAPK and Akt pathway in kidneys of burn rats.

## Methods

### Animal model

This study was carried out in strict in accordance with the National Institutes of Health Guidelines for the Use of Laboratory Animals. The animal protocol was approved by the Fourth Military Medical University Committee on Animal Care (Permit Number: 12008). Male Sprague-Dawley rats (175–225 g) were anesthetized with sodium pentobarbital (60 mg/kg intraperitoneally) as the general anesthesia and 2% lidocaine with 1∶100000 epinephrine as the local anesthesia, and subjected to a full-thickness scald burn [28]. Briefly, the back and flank skin were shaved, and the animal was secured into a specially designed template. The skin was immersed in 100°C water for 15 s to produce a full thickness dermal burn over 40% of the total body surface area (TBSA). Sham-operated rats were treated in a manner identical to the burn treatment except exposing to room-temperature (25°C) water and received no study drug (n = 6). After immersion, all the animals were then resuscitated with lactated Ringer solution (intraperitoneal injection at 4 ml/kg immediately and 8 h after burn). The breath and heart rate of burn rats were carefully monitored to ensure that all rats were under anesthetic and painless before the rats were recovered from anesthesia. The rats subjected to burn were randomized to receive one of the following solutions: 1) vehicle (0.9% NaCl, intravenous infusion at 4 ml/kg); 2) tempol (Sigma, St. Louis, MO): a synthetic antioxidant and a superoxide dismutase (SOD) mimetic (intravenous infusion at 7.5 mg/ml/kg) [29]; 3) SB203580 (Sigma, St. Louis, MO): p38 MAPK inhibitor (intraperitoneal injection at 7.5 mg/ml/kg) [30]; 4) Akt inhibitor IV (Calbiochem, San Diego, CA) (intraperitoneal injection at 0.03 mg/ml/kg [31]. These treatments were given within 10 min after burn and continued every 12 h until the rats were sacrificed by overdose of sodium pentobarbital and tissues collected for the biochemical and histological measurements. Animals in vehicle treatment group were culled at 3 h, 6 h, 24 h, 48 h and 72 h after burn (n = 6 for each time point) respectively, while those receiving tempol, SB203580 or Akt inhibitor treatment were culled at 72 h after burn (n = 6 for each treatment). Tertiary butyl hydroperoxide (tBHP) (Sigma, St. Louis, MO) was used (intraperitoneal injection at 2.5 mmol/kg/day) to generate redox condition in sham rats (n = 6), and kidney tissues were collected after treatment with tBHP for 2 weeks [32].

### Renal function monitoring

Blood samples for determination of renal function were obtained at different timepoints and serum levels of creatinine and blood urea nitrogen (BUN) were measured using a clinical chemistry analyzer system and kits (Prochem-V, Drew Scientific, Dallas, TX).

### Histological examination for tubular damage

Fixed kidneys embedded in paraffin were sectioned at 6 μm thickness and periodic acid–Schiff (PAS) staining was performed on tubular damage under the microscope (200× magnification). Histological changes were graded in a blinded manner by two observers and scored based on the percentage of cortical tubules showing epithelial necrosis: 0, normal; 1, >10%; 2, 10 to 25%; 3, 26 to 75%; and 4, <75%. Tubular necrosis was defined as the loss of the proximal tubular brush border, blebbing of apical membranes, tubular epithelial cell detachment from the basement membrane, or intraluminal aggregation of cells and proteins [33].

### Detection of apoptosis by TUNEL and caspase-3

Paraffin sections were dewaxed and in situ detection of apoptosis in the renal tissues was performed by TUNEL (terminal deoxynucleotidyl transferase dUTP nick end labeling) assay according to the instructions of the manufacturer (Roche Molecular Biochemicals, Mannheim, Germany). A double-staining technique was used, i.e., TUNEL staining for apoptotic cell nuclei and 4′,6-diamino-2-phenylindole staining for all cell nuclei. The index of apoptosis was expressed by number of apoptotic cells/the total number of cells counted ×100% and the assays were performed in a blinded manner. Caspase-3 activation in tissue was measured using western blot.

### Determination of H_2_O_2_, TBARS, total SOD and manganese SOD (MnSOD) content in kidney

H_2_O_2_ in renal tissues was measured using an Amplex Red Hydrogen Peroxide/Peroxidase Assay kit (Molecular Probes, Invitrogen, Carlsbad, CA) according to the manufacturer's instructions. In the presence of peroxidase, the Amplex Red reagent reacts with H_2_O_2_ in a 1∶1 stoichiometry to produce a red-fluorescent oxidation product, which is assessed fluorometrically with 530 nm excitation and 590 nm emission wavelengths. Malondialdehyde, measured as thiobarbituric acid-reactive substances (TBARS), is a secondary product of lipid peroxidation. The concentration of TBARS in renal tissue was analyzed with TBARS Assay Kit (Cell Biolabs, San Diego, CA) according to the manufacturer's instructions. The total SOD activity in renal tissue was determined by monitoring the rate of reduction of nitroblue tetrazolium using a xanthine–xanthine oxidase system as the source of O_2_ with a SOD assay kits (R&D Systems, Minneapolis, MN, USA). SOD activity in the experimental renal tissue was measured as the percent inhibition of the rate of formazan dye formation [28]. A commercially available kit (Jiancheng Biotech Inc., Nanjing, China) was used to measure the MnSOD activity in renal extracts according to the manufacturer's instructions. Potassium cyanide was used to inhibit CuZnSOD, resulting in the detection of the MnSOD activity only.

### Western blot analysis

All the antibodies were obtained from Cell Signaling Technology (Santa Cruz, CA). Frozen kidney tissue samples were cut into pieces and lysed with RIPA lysis buffer for 10 min on ice. After sonication, the lysates were centrifuged at 12000 g for 5 min and the proteins were extracted. The kidney protein samples were mixed in loading buffer, boiled for 10 min, and then subjected to SDS–PAGE. Proteins were transferred onto nitrocellulose membranes by electrophoresis and then were incubated overnight at 4°C with the following primary antibodies (1∶1,000): anti-p-p38, anti-p-ERK, anti-p-JNK, anti-p-Akt, anti-Nox4, anti-MnSOD, anti-β-actin, anti-glyceraldehyde-3-phosphate dehydrogenase (GAPDH). Membranes were washed with PBS-T and incubated with a secondary antibody at room temperature for 1 h. Protein bands were visualized by ECL-Plus reagent (GE Healthcare, Piscataway, NJ). The p-p38, p-ERK, p-JNK or p-Akt immunoblots were then stripped with strip buffer at 50°C for 30 mins and reblotted for total p38, ERK, JNK or Akt. Results were normalized to GAPDH or β-actin.

### Statistical analysis

Results are presented as mean ± SEM. Data were analyzed by one-way ANOVA followed by Bonferroni's multiple comparison test using GraphPad Prism software (San Diego, CA, USA). A value of *P*<0.05 was considered to be significant.

## Results

### Burn injury caused late acute renal failure in rats

**Figure 1:**
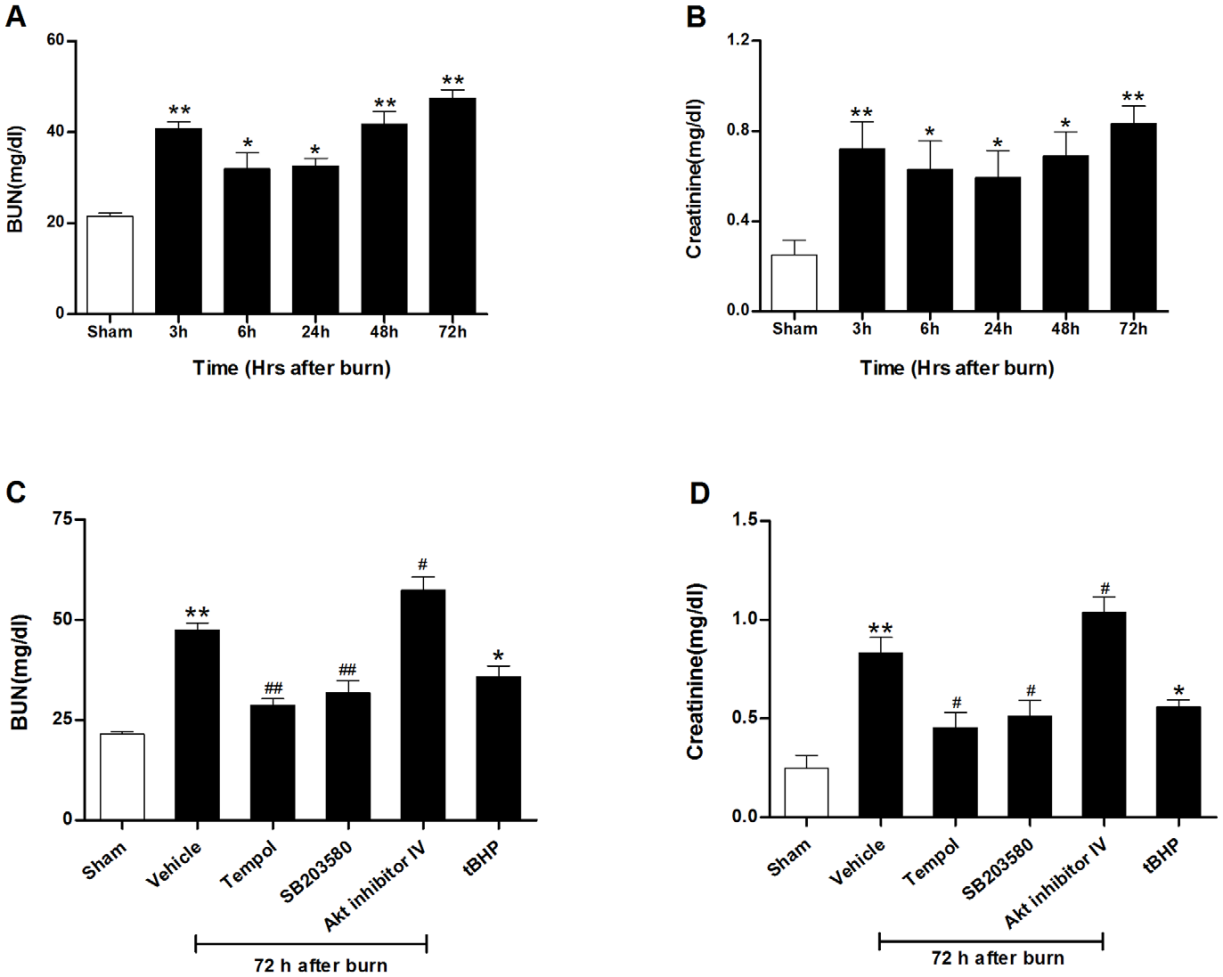
Blood urea nitrogen (BUN) and creatinine in rats after burn. Analysis of burn-induced late renal dysfunction evidenced by BUN and creatinine (**A–B**) at different time points of 3 h, 6 h, 24 h, 48 h and 72 h and (**C–D**) with different treatments at 72 h postburn. The data showed that burn induced a late renal dysfunction at 72 h, which was improved by inhibiting reactive oxygen species (ROS) or p38 MAPK with tempol or SB203580, and deteriorated by inhibiting Akt expression at 72 h. Treatment with tBHP also caused significant renal dysfunction. *P<0.05, **P<0.01 vs. Sham; ^#^ *P*<0.05, ^##^ *P*<0.01 vs. Vehicle. n = 6 rats per group per time point.

To investigate the time stage of burn-induced renal dysfunction, levels of BUN and serum creatinine were measured at 3 h, 6 h, 24 h, 48 h and 72 h after burn. As shown in Fig. 1A–B, both BUN (40.8±1.5 vs. 21.6±0.7 mg/dl in sham-burned group; *P*<0.01) and creatinine (0.72±0.12 vs. 0.25±0.07 mg/dl in sham-burned group; *P*<0.01) increased significantly at 3 h and then decreased (32.6±1.7 mg/dl of BUN and 0.59±0.12 mg/dl of creatinine) during the first 24 h after burn. However, both BUN and creatinine went up again at 48 h after burn and peaked at 72 h (47.5±1.8 mg/dl of BUN and 0.83±0.08 mg/dl of creatinine), suggesting both early (3 h) and late (72 h) renal dysfunction occurred in our experimental burn injury model.

**Figure 2:**
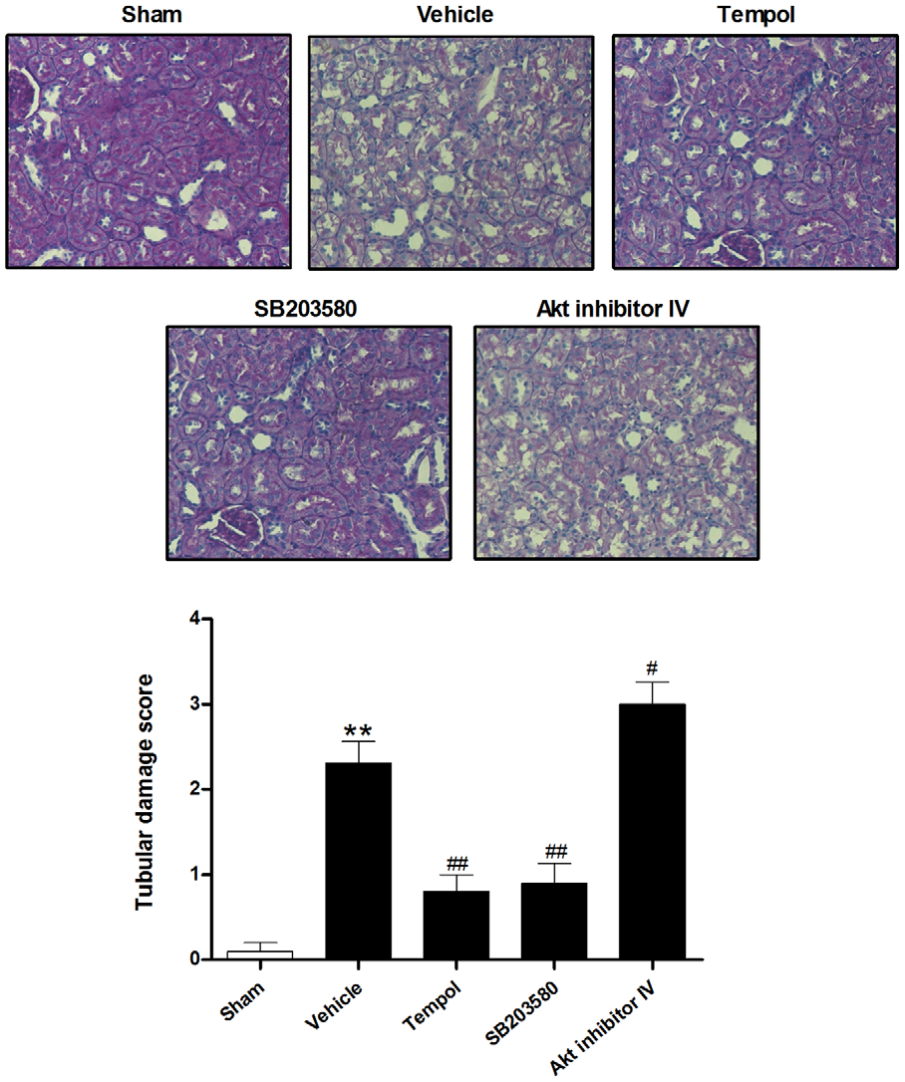
Histopathological renal injury in rats at 72 h after burn. Representative images (magnification  = 200×) showed the histological examination for tubular damage with different treatments postburn using periodic acid–Schiff (PAS) staining. The data indicated burn induced significant renal damage at 72 h, which was improved by inhibiting ROS production or p38 MAPK expression, and deteriorated by inhibiting Akt expression. **P<0.01 vs. Sham; ^#^ *P*<0.05, ^##^ *P*<0.01 vs. Vehicle. n = 6 rats per group per time point.

Furthermore, histological examination revealed burn injury caused necrosis, vacuolation, and desquamation of epithelial cells in the renal tubules of the vehicle-treated rats with higher tubular damage score (2.3±0.3; *P*<0.01) than sham-burned group (0.1±0.1) at 72 h after burn (Fig. 2). All these results above indicated that severe burn injury (40% of TBSA) resulted in significant late renal dysfunction and tubular damage at 72 h in rats.

### Burn injury induced renal tubular cell apoptosis

**Figure 3:**
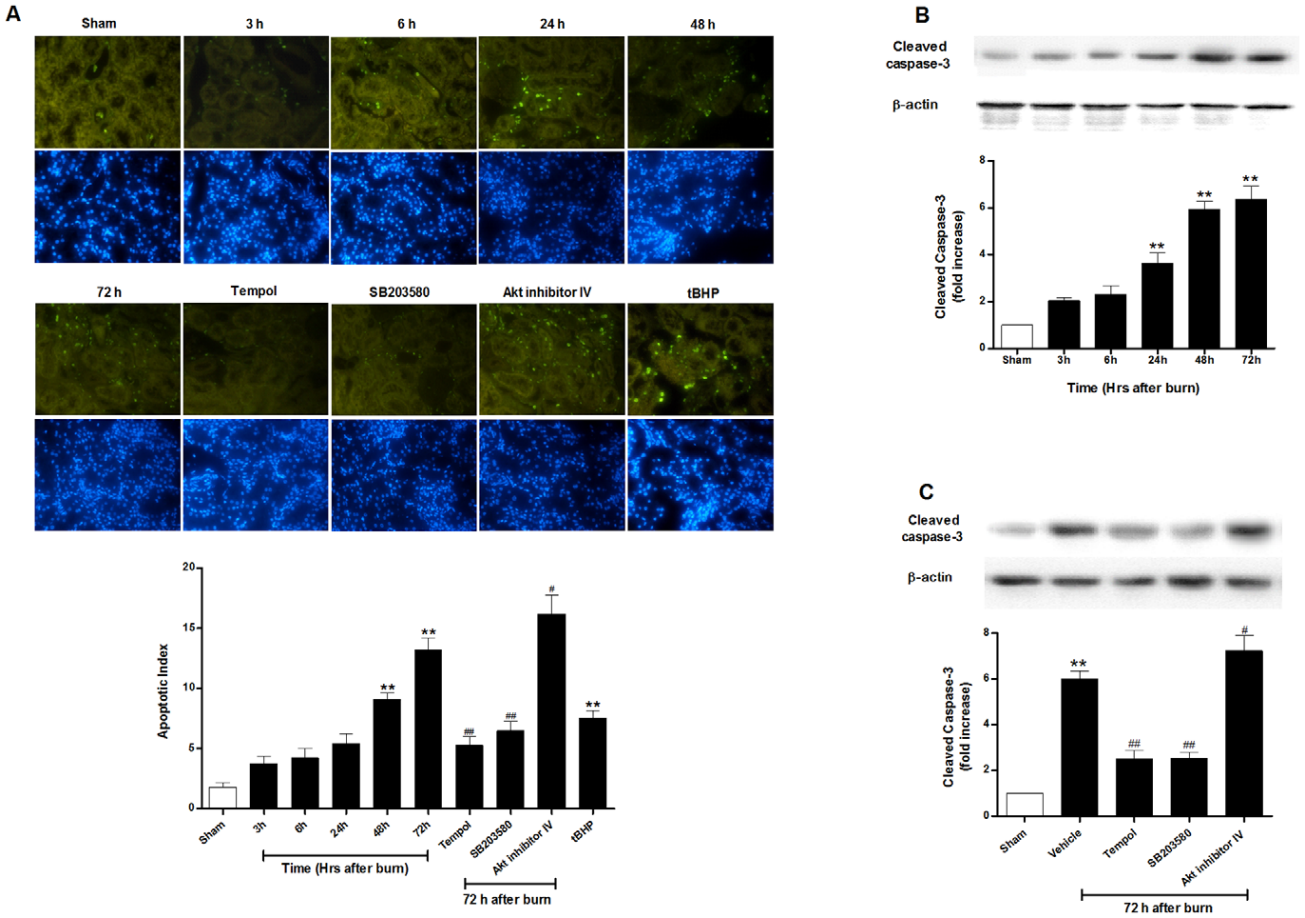
Tubular cell apoptosis and caspase-3 activation in rats after burn. Burn induced (**A**) tubular apoptosis presented by renal TUNEL staining (magnification  = 400×) and increased caspase-3 activation (**B**) at different time points of 3 h, 6 h, 24 h, 48 h and 72 h (**C**) with different treatments at 72 h postburn. The data showed that burn induced increased caspase-3 activation and tubular cell apoptosis at a relatively late time stage postburn. Inhibition of ROS production and p38 MAPK phosphorylation alleviated renal apoptotic injury, while blockage of Akt expression aggravated the apoptosis in late renal failure after burn. Administration of tBHP led to obvious apoptotic kidney injury. **P<0.01 vs. Sham; ^#^ *P*<0.05, ^##^ *P*<0.01 vs. Vehicle (72 h). n = 6 rats per group per time point.

TUNEL staining showed that tubular apoptotic cells increased slightly and insignificantly during the first day after burn (all *P*>0.05), but grew up rapidly after 24 h and continually rose to 72 h (14.5±1.2% vs. 1.4±0.3% in sham-burned group; *P*<0.01) (Fig. 3A).

As shown in Fig. 3B, burn injury induced significant caspase-3 activation from 24 h after burn and gradually elevated afterwards. More than 5-fold increase was observed in burned rats at 72 h compared with sham-burned group (*P*<0.01). All these results above indicated severe burn induced obvious apoptotic kidney injury in rats at late stage after burn.

### Burn-induced sustained ROS generation contributed to tubular apoptosis and renal dysfunction

**Figure 4:**
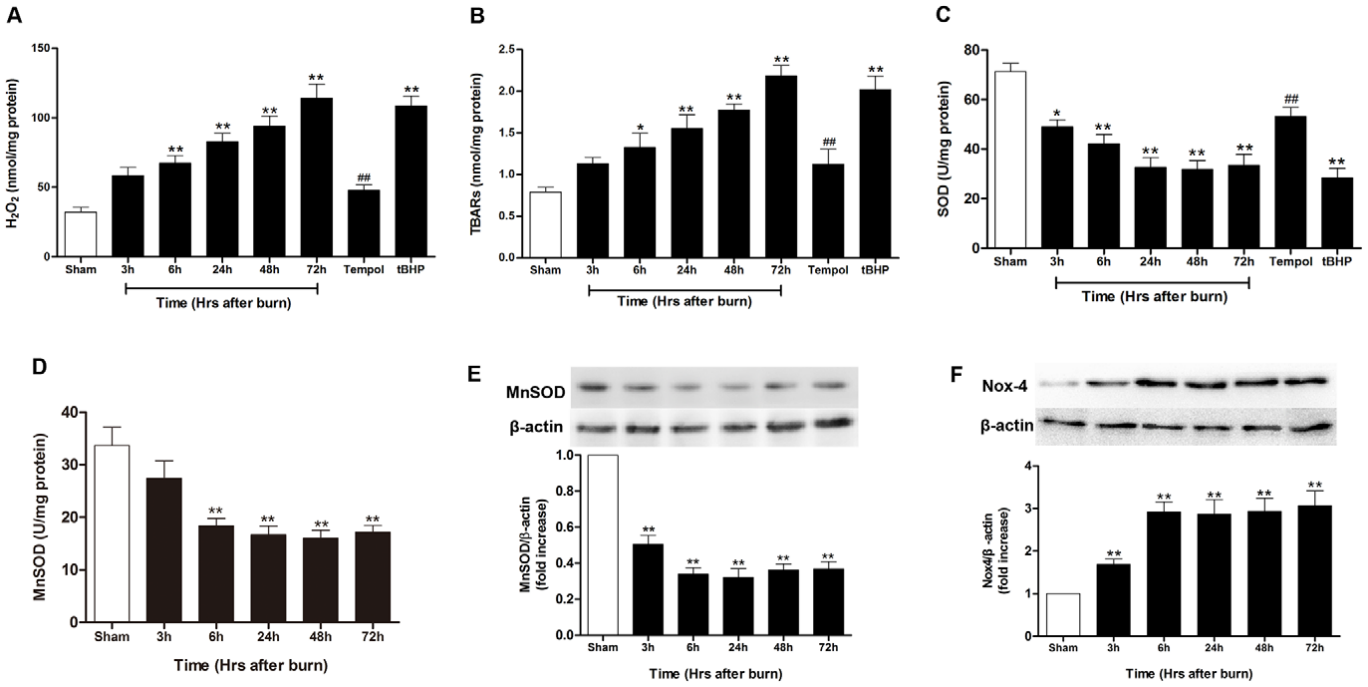
Induction of oxidative stress in kidneys of rats postburn. Burn injury induced sustained ROS generation evidenced by increased renal (**A**) H_2_O_2_ production, (**B**) thiobarbituric acid-reactive substances (TBARS) generation, suppressed antioxidant activity evidenced by decreased (**C**) SOD level and at 3 h, 6 h, 24 h, 48 h and 72 h after burn, which were attenuated by tempol at 72 h after burn. Renal (**D**) MnSOD activity and (**E**) protein expression, and (**F**) Nox4 expression was analyzed to determine the mitochondrial status. Treatment with tBHP also induced significant oxidative stress in kidneys of sham rats. *P<0.05, **P<0.01 vs. Sham; ^##^ *P*<0.01 vs. Vehicle (72 h). n = 6 rats per group per time point.

ROS was respectively measured using H_2_O_2_ and TBARS in the kidneys of rats exposed to burn. Compared with sham-burned group (32.0±3.6 nmol/mg protein), renal cell H_2_O_2_ production markedly increased in the burn rats at 6 h (67.4±5.4 nmol/mg protein; *P*<0.01) and continually elevated to 72 h (114.0±10.1 nmol/mg protein; *P*<0.01) postburn (Fig. 4A). Tissue TBARS was significantly higher beginning at 6 h (1.33±0.17 nmol/mg protein; *P*<0.05) and sustained up to 72 h (2.19±0.13 nmol/mg protein; *P*<0.01) postburn compared with sham-burned group (0.79±0.06 nmol/mg protein) (Fig. 4B). In order to further evaluate the redox system state the possible role of mitochondrial ROS in burn-induced ARF, the total SOD activity and the protein expression and activity of MnSOD were detected and the data showed that total SOD activity reduced (49.0±2.8 U/mg protein vs. 71.3±3.4 U/mg protein in sham-burned group, *P*<0.05) at 3 h after burn injury, continued to decrease until 24 h postburn (32.7±3.9 U/mg protein), and remained at low level thereafter (Fig. 4C). As shown in Fig. 4D–E, the MnSOD activity significantly decreased at 6 h, 24 h, 48 h and 72 h after burn (*P*<0.01), while renal MnSOD expression significantly decreased at 3 h after burn, and stayed at low level during 72 h post-injury (*P*<0.01). In addition, the Nox4 expression, which was one of the mitochondrial complex that indicated mitochondrial status, was analyzed by western blot to determine the potential sources of the radicals. The results (Fig. 4D) showed that Nox4 expression in kidney increased at 3 h postburn and during the whole 72-h experiment it was significantly higher than that of sham-burned group (P<0.01). All these data showed that severe burn injury (40% of TBSA) induced a sustained renal ROS production in rats during 72 h after burn, which may derived from the nicotinamide adenine dinucleotide phosphate reduced form (NADPH) oxidase.

Compared with the vehicle-treated group, administration of tempol within 10 min after burn and continued every 12 h during the whole experiment markedly reduced ROS generation and increased SOD activity (Fig. 4), significantly decreased BUN (28.6±1.7 mg/dl; *P*<0.01) and creatinine (0.45±0.08 mg/dl; *P*<0.05) (Fig. 1C–D), and improved tubular damage (0.8±0.2; *P*<0.01) at 72 h (Fig. 2). Moreover, tempol treatment significantly inhibited TUNEL-positive tubular cells (5.3±0.7%; *P*<0.01) (Fig. 3A) and caspase-3 activation (Fig. 3C) in kidney at 72 h. In addition, use of tBHP in sham rats induced oxidative stress evidenced by increased renal H_2_O_2_ concentration (108.5±7.1 nmol/mg protein), TBARS level (2.02±0.16 nmol/mg protein) and decreased SOD level (28.3±3.9 U/mg protein) as compared to the sham-burned group (all *P*<0.01) (Fig. 4A–C), and thus led to significant tubular cell apoptosis (7.4±0.8%; *P*<0.01) (Fig. 3A) and renal dysfunction as assessed by elevated BUN (35.9±2.6 mg/dl; *P*<0.05) and creatinine (0.56±0.04 mg/dl; *P*<0.05) (Fig. 1C–D). These data indicated that sustained ROS overproduction may contribute to tubular cell apoptosis and late renal dysfunction after burn.

### Changes of p38 MAPK and Akt phosphorylation in kidney postburn

**Figure 5:**
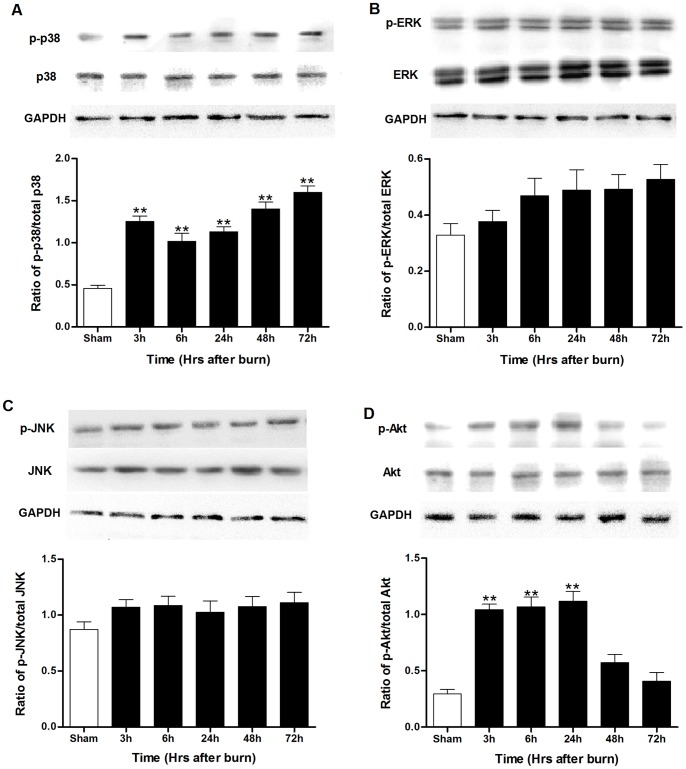
Expression of MAPK and Akt signaling in kidneys of rats postburn. Burn induced (**A–C**) elevated renal p38 MAPK phosphorylation in a time dependent manner but had no significant effect on ERK or JNK, whereas (**D**) Akt phosphorylation increased during the first 24 h but decreased beginning from 48 h after burn. **P<0.01 vs. Sham. n = 6 rats per group per time point.

It has been reported that MAPK signaling is involved in renal dysfunction after burn injury [34], so we measured the phosphorylation of p38, JNK and ERK1/2 MAPKs in rat kidneys at 3 h, 6 h, 24 h, 48 h and 72 h after burn. As shown in Fig. 5A–C, p38 MAPK phosphorylation elevated at 3 h, dropped a little at 6 h, increased again at 24 h and reached a peak level at 72 h postburn, while JNK phosphorylation did not change at any time point during the whole 72-h experiment. There was slight but insignificant increase in ERK phosphorylation after burn. No difference in total p38, JNK or ERK1/2 MAPKs protein abundance was observed between burn injury and sham-burned groups.

As Akt pathway is the major anti-apoptotic pathways that confer the survival advantage, we also investigated Akt phosphorylation in rat kidneys at 3 h, 6 h, 24 h, 48 h and 72 h after burn. The results in Fig. 5D demonstrated that Akt phosphorylation was up-regulated at 3–6 h after burn and reached a peak at 24 h. It is worth noting that Akt phosphorylation reduced at 48 h and returned to an almost normal level at 72 h postburn compared with sham-burned group, which exerted a similar temporal variation with renal dysfunction postburn. There was no difference in Akt protein abundance between these two groups.

### Duplex Regulation on p38 MAPK and Akt Phosphorylation by ROS

Having observed that p38 MAPK and Akt phosphorylation exerted different temporal variation in the 72 hours after burn injury, we next verified the possible roles of p38 and Akt phosphorylation in renal apoptosis and dysfunction. The rats were treated with the p38 inhibitor SB203580 or Akt inhibitor IV immediately after burn injury. SB203580 treatment inhibited tubular apoptosis as evidenced by reduced TUNEL-positive staining cells (Fig. 3A) and cleaved caspase-3 expression (Fig. 3C), decreased tubular damage (Fig. 2), led to an improved renal function at 72 h after burn (Fig. 1C–D). In contrast, treatment with Akt inhibitor IV further increased renal cell apoptosis (Fig. 3A and Fig. 3C) and necrosis (Fig. 2) and thus deteriorated renal dysfunction 72 h after burn injury (Fig. 1C–D).

**Figure 6:**
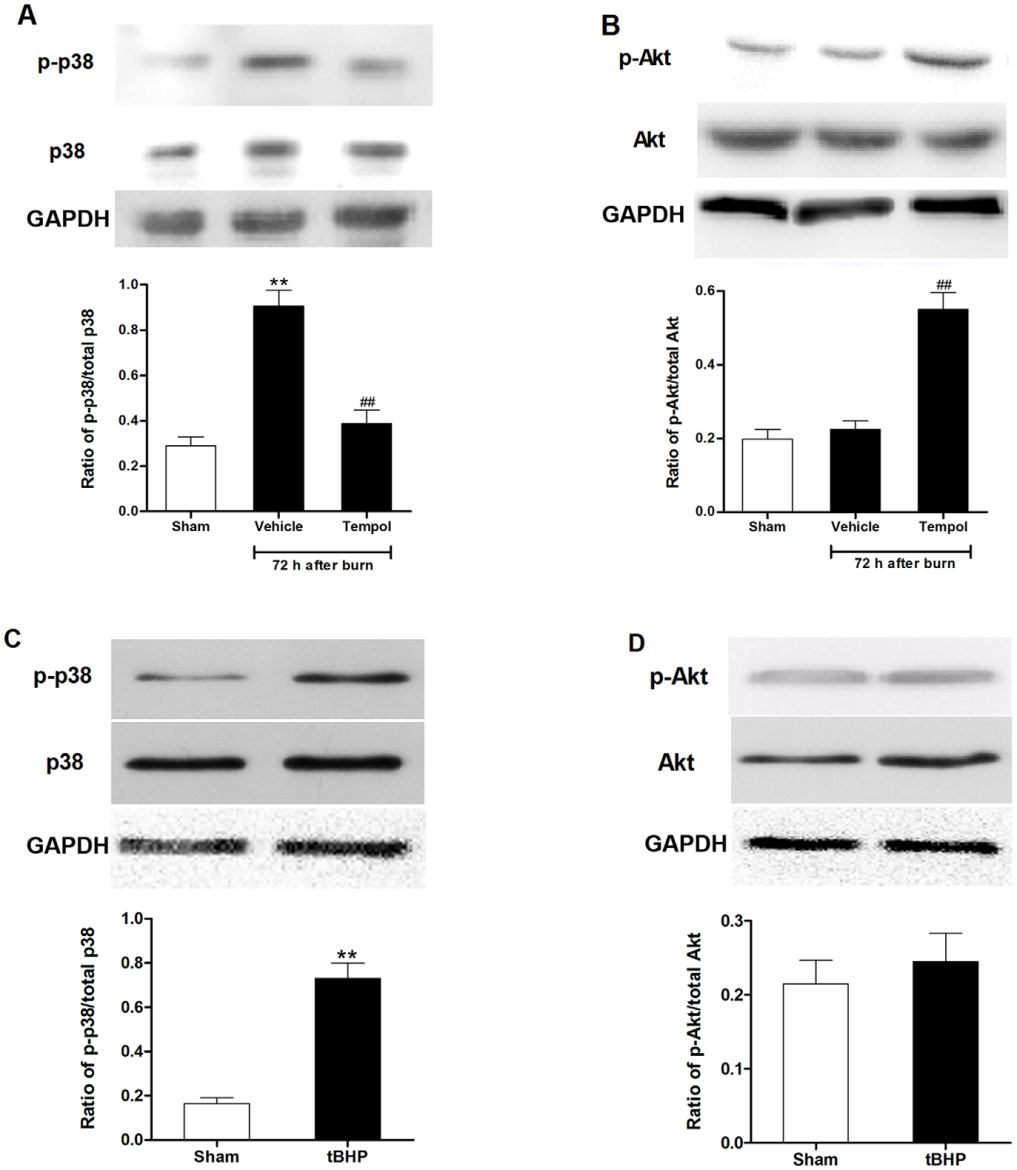
Effect of ROS on expression of MAPK and Akt in kidneys of rats postburn. (**A**–**B**) Tempol attenuated ROS-induced phosphorylation of p38 and inhibition of Akt at 72 h postburn. (**C**–**D**) Induction of ROS with tBHP significantly increased p38 but inhibited Akt phosphorylation. **P<0.01 vs. Sham; ^##^ *P*<0.01 vs. Vehicle. n = 6 rats per group.

**Figure 7:**
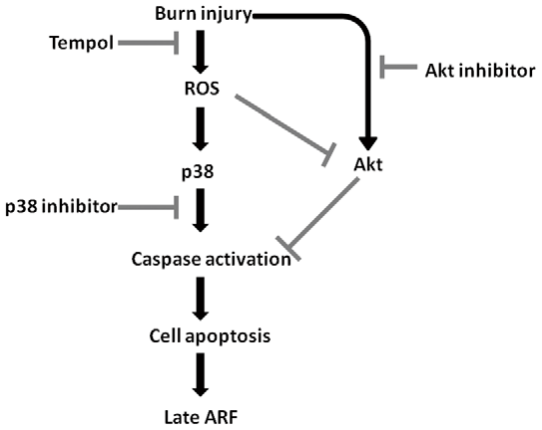
Schematic diagram showed the mediation of burn-induced sustained ROS overproduction on p38 MAPK and Akt expression and apoptotic pathway, which may be the mechanism of late acute renal failure.

Interestingly, administration of tempol significantly down-regulated p38 MAPK phosphorylation at 72 h, whereas up-regulated Akt phosphorylation at 72 h after burn (Fig. 6A–B). Induction of ROS by tBHP also significantly increased the expression of phosphorylated p38, however, with insignificant changes in the expression of pAkt (Fig. 6 C–D). All these results suggested that sustained ROS overproduction may increase p38 MAPK phosphorylation but further inhibit Akt phosphorylation at 72 h postburn, and thus resulted in tubular cell apoptosis and late ARF after burn (Fig. 7).

## Discussion

Two salient observations were made from the present study. First, sustained renal ROS production during 72 hours after burn caused continuous tubular apoptosis and thus induced late renal dysfunction in severely burned rats. Second, scavenging burn-induced ROS overproduction inhibited p38 MAPK phosphorylation but further increased Akt phosphorylation which reduced cell apoptosis and protected renal function against burn injury.

Severe ARF, although occurring in the minority of burn patients, repeatedly was shown to have devastating consequences in this setting and associated with extremely high mortality [1], [35], [36]. It is reported that the late onset ARF, which begins 5 days post-burn, becomes the most frequent cause of renal insufficiency in burn patients nowadays [2]. These reports are consistent with our data that after suffering from immediate renal dysfunction, the rats recovered a little at 6 h but showed more severe kidney injury evidenced by deteriorative renal dysfunction and tubular damage 3 days after burn injury, revealing that a late ARF was induced by burn injury. As a higher incidence of hypotension has been found during the resuscitation phase in patients with early ARF, the prognosis developing of late ARF in burn patients is multifactorial and involved in sepsis, administration of antibiotics and cytokine response [4], [5].

Several common renal insults cause apoptosis in the kidney, including ischemia, toxic injury, radiation, and burn [37], [38], [39]. A recent clinical study reported that plasma-induced apoptosis on tubular cells was related to the severity of renal dysfunction and patient prognosis after burn [40]. The present study demonstrated that burn injury did not result in primary apoptotic cell death in the kidney during the first day postburn, whereas caused the increasing apoptosis in renal tubular cells in the late procedure, in parallel with late renal dysfunction after burn. This delayed apoptosis was reported to contribute to the secondary organ injury after severe stimuli [41]. So therapeutic interventions that effectively inhibit apoptosis have potential for minimizing renal dysfunction and accelerating recovery after ARF [42].

Inordinate or aberrant generation of ROS is widely incriminated in the pathogenesis of burn-induced tissue injury [10]. The kidney has long been studied as an organ that can generate ROS and is vulnerable to the damaging effects of ROS [12], [43]. A series of reports demonstrated that following a burn, enormous ROS was produced which was harmful and implicated in renal cell death and tissue damage [44], [45]. Similar with these studies, we found increased formation of ROS in kidneys of burn rats. Although ROS induction was always proposed to happen immediately after stress, the data in our study showed the generation of ROS began at 3 h after burn and continued to rise up to 72 h post-burn. A recent study confirmed our results of sustained ROS formation till 72 h after burn, which attributed to defects in ROS defense including loss of membrane integrity and antioxidant defense in mitochondria [46]. As SOD is one of main enzymatic antioxidants for scavenging superoxide, and thus relieve the cellular stress caused by superoxide anion and improve organ function under disease conditions [17], [47], the significant downregulation of kidney SOD activity after burn injury may contributed to the burn-mediated persistent oxidative damage to renal.

In the present study, use of antioxidant intervention with tempol significantly scavenged ROS and alleviated delayed renal injury, while administration of tBHP induced obvious oxidative stress in kidney and resulted in renal dysfunction, manifesting the main contribution of ROS to burn-induced late renal failure. Interestingly, treatment with tempol also attenuated burn-induced kidney apoptosis. It is well known that ROS can induce apoptosis in a variety of cells including renal tubule cells in response to a wide variety of apoptotic stimuli including hypoxia, ischemia/reperfusion and drug injury [48]. Excessive intracellular ROS was suggested to cause renal apoptosis of tubular epithelial cells via triggering a variety of apoptotic mediators [42]. All these data above provide the first evidence for the apoptotic role of persistent ROS production in late ARF after severe burn injury.

Many enzymatic systems implicated in ROS generation in the kidney, including mitochondrial respiration, uncoupled eNOS, and NADPH oxidase (Nox) [49]. Among them, Nox, especially Nox4, may be an oxygen sensor that regulates erythropoietin production in the kidney, and it has been implicated as a major source of renal ROS [50]. Recent studies showed that Nox4 localizes in mitochondria and that, in the animal model of diabetes [11], ischemic stroke [51] and nephrotoxicity drugs [33], mitochondrial Nox4 expression is increased. In the present study, we found that severe burn injury induced increased expression of Nox4, which may contribute to the ROS overproduction in renal. In addition, recent studies have shown that the MnSOD, which is the major mitochondrial antioxidant responsible for scavenging superoxide radicals generated by the respiratory chain activity or via mitochondrial stressors, was inactivated in renal disorders further suggesting that loss of MnSOD activity was a key event in renal damage [52], [53]. The downregulation of MnSOD and loss of MnSOD activity in our study could lead to increased levels of superoxide within the mitochondria, which may result in ultimately mitochondrial dysfunction and cell apoptosis in burn-induced ARF. Further investigations are in need to elucidate the molecular mechanism of potential sources of the renal radicals after burn, which may lead to targeted oxidative stress control at early post-burn stage for better kidney protection in burned patients.

As important transducers of cell signaling, activation of MAPK was essential in causing renal apoptosis injury and the inhibition of MAPK activation blocked the development of the renal injury [54]. We assessed the p38, ERK and JNK MAPK expression in kidney which showed that burn treatment obviously changed p38 expression, however, with only slight increase in ERK phosphorylation and no obvious effect on JNK phosphorylation. Furthermore, treatment with SB203580 restrained p38 phosphorylation and in turn decreased tubular cell apoptosis and improved renal function, indicating the p38 pathway but not ERK or JNK played an important role in ARF after burn.

The p38 families were described in the setting of cell response to stress and reported to increase immediately after burn injury [54], [55]. However, Hiroaki Sato et al demonstrated that activation of p38 MAPK in acute phase after stress was highest in 1–3 h and decreased gradually at 5 h [56]. Similar results were found in the present study, whereas a re-activation of p38 was observed 48 h after burn. ROS, which derived from a number of cellular stimuli, has been demonstrated to induce or mediate the activation of the MAPK pathways [57]. We found that burn injury induced ROS production also in parallel with the secondary elevation of p38 phosphorylation after the first day postburn, which was blocked by treatment with tempol at 72 h after burn. In addition, induction of ROS accumulation with tBHP increased the phosphorylation of p38, indicating the involvement of persistent ROS generation in re-activation of p38 pathways 72 h postburn.

Previous research reported the activated Akt which was a cell survival regulation pathway could protect rat kidney from apoptosis by enhancing antioxidant capacity, and reducing apoptotic proteins content [58]. Our study showed that phosphorylation of Akt elevated in the first day after burn but gradually disappeared as time went on. The decrease of p-Akt aggravated tubular apoptosis and worsened renal function 72 h after burn. This phenomenon was in line with the data of an animal study showing that the elevated pAkt level gradually returned to normal from 12 h after burn. Recent studies demonstrated that the high levels of mitochondrial ROS down-regulated phosphoinositide 3-kinase (PI3K)–Akt signaling [27]. In the present study, the usage of tempol markedly reverted the Akt activation 72 h post-burn, while no obvious increase in pAkt was found with administration of tBHP for 2 weeks, indicating sustained ROS may contribute to the depressed Akt phosphorylation at late stage after burn.

### Conclusion

In summary, we have demonstrated that sustained renal ROS overproduction after burn induced continuous tubular cell apoptosis and thus a late ARF, which may result from ROS-mediated activation of p38 MAPK but a late inhibition of Akt phosphorylation in severely burned rats, and that anti-oxidative treatment may represent a promising potent therapy in clinical applications of late ARF after burn. These results may also have important implications for the treatment of other kidney diseases associated with apoptotic injury and interrelated oxidative stress.
