# Expression of Concern: Sustained Oxidative Stress Causes Late Acute Renal Failure via Duplex Regulation on p38 MAPK and Akt Phosphorylation in Severely Burned Rats

**DOI:** 10.1371/journal.pone.0298294

**Published:** 2024-02-01

**Authors:** The PLOS ONE Editors

After this article [1] was published, concerns were raised about results presented in Figs 2–5. Specifically:

- The Tempol panel in Fig 2 appears to partially overlap with the SB203580 panel in Fig 2.
- In the Cleaved caspase-3 panel in Fig. 3B, when levels are adjusted to visualize background, lane 1 appears to be discontinuous with adjacent regions.
- There appears to be a vertical discontinuity between lanes 5 and 6 in the β-actin panel in Fig. 4F.
- The β-actin panel in Fig. 3C appears similar to lanes 2–6 in the JNK panel in Fig. 5C.
- The p38 panel in Fig. 5A appears similar to the Akt panel in Fig. 5D.

Individual-level data for the charts in Figs 2–5 from the time of the original experiments are provided here in S1 File. The first author stated that the individual-level quantitative data underlying Figs. 1 and 6 are available upon request but that the underlying uncropped and unadjusted blots for all published blots are no longer available. The above concerns about results presented in Figs. 3–5 are therefore unresolved.

In response to queries about the experiments in Fig 2, the first author stated that the SB203580 panel is incorrect. They provided an updated version of Fig 2 in which the SB203580 panel has been corrected. The underlying images from the original experiments, replicate images from later repeat experiments, and individual-level quantitative data for Fig 2 are provided here in S1–S2 Files. The original data underlying the Tempol and SB203580 panels in Fig 2 (S2 File) have been reviewed by PLOS.

In response to queries about the experiments in Fig. 3B, the first author stated that there were seven lanes in the original blot for Fig. 3B, and a lane was removed between lanes 1 and 2 in the published cleaved caspase 3 and β-actin panels. In the absence of original uncropped images for this figure, this issue remains unresolved.

Replicate underlying images and individual-level quantitative data for Figs. 3–5 from later repeat experiments are provided here in S2-S3 Files.

In light of the unresolved concerns in Figs. 3–5, the extent of image issues in this article, and the unavailability of the original underlying image data for the figures of concern, the *PLOS ONE* Editors issue this Expression of Concern.

**Fig 2:**
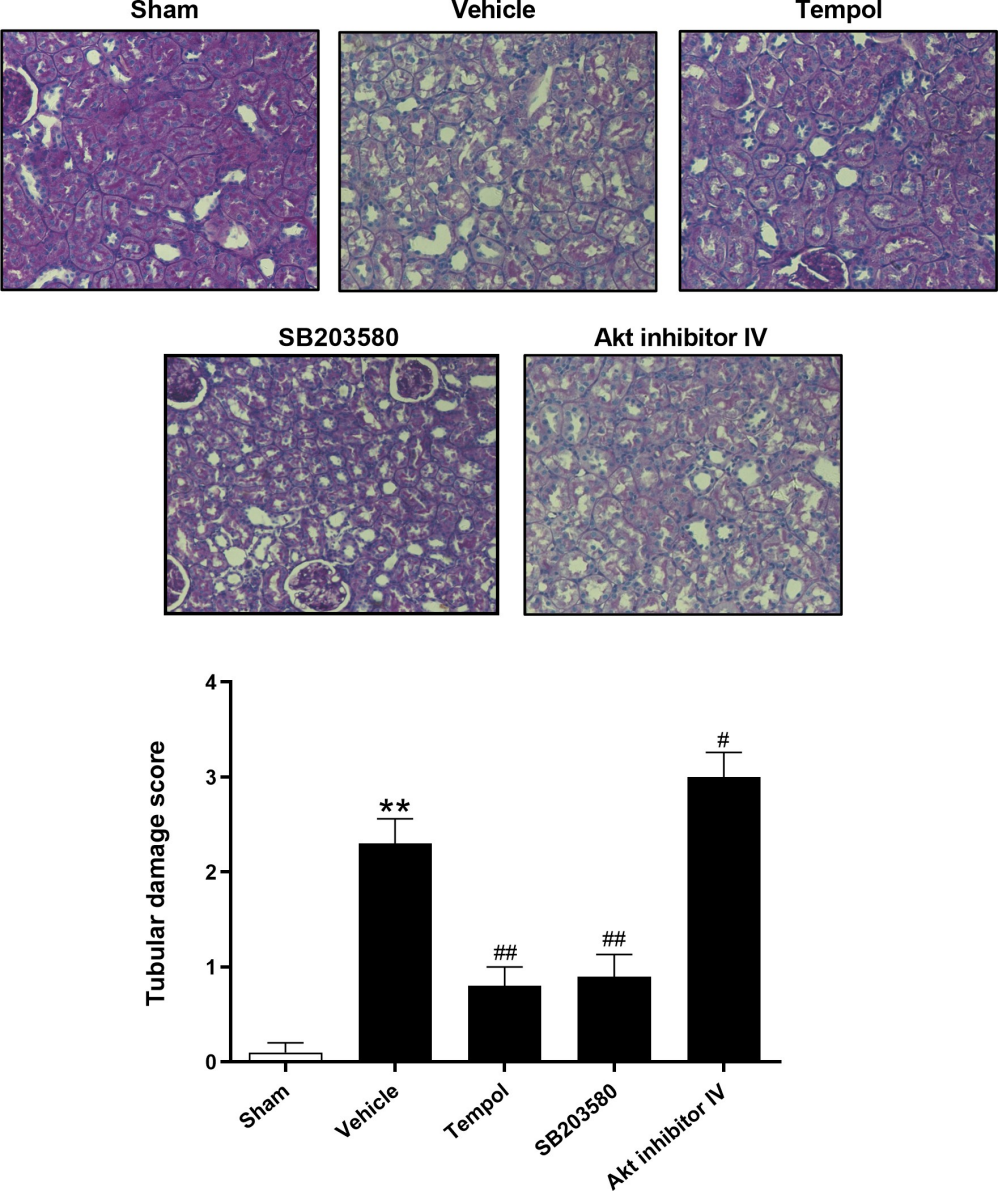
Histopathological renal injury in rats at 72 h after burn. Representative images (magnification = 200 x) showed the histological examination for tubular damage with different treatments postburn using periodic acid–Schiff (PAS) staining. The data indicated burn induced significant renal damage at 72 h, which was improved by inhibiting ROS production or p38 MAPK expression, and deteriorated by inhibiting Akt expression. **P<0.01 vs. Sham; #P<0.05, ##P<0.01 vs. Vehicle. n = 6 rats per group per time point.

## Supporting information

S1 File Individual-level data for the charts in Figs 2–5 from the time of the original experiments. (RAR)

S2 File Underlying original and replicate histological images from later repeat experiments for Fig 2, underlying original images for Figure 3A, and underlying replicate blots from later repeat experiments for Figures 3B-C, 4E-F and 5. (RAR)

S3 File Individual-level data for the charts in Figures 3A and 4A-D from the time of the original experiments and replicate individual-level data for the charts in Figures 3B-C and 4E-F and 5 from later repeat experiments. (RAR)

## References

[1] Feng Y, Liu Y, Wang L, Cai X, Wang D, Wu K, et al. (2013) Sustained Oxidative Stress Causes Late Acute Renal Failure via Duplex Regulation on p38 MAPK and Akt Phosphorylation in Severely Burned Rats. PLoS ONE 8(1): e54593. doi: 10.1371/journal.pone.0054593 23349934 PMC3547934

